# Control Measurements of Crane Rails Performed by Terrestrial Laser Scanning

**DOI:** 10.3390/s17071671

**Published:** 2017-07-20

**Authors:** Klemen Kregar, Jan Možina, Tomaž Ambrožič, Dušan Kogoj, Aleš Marjetič, Gašper Štebe, Simona Savšek

**Affiliations:** 1 Faculty of Civil and Geodetic Engineering, University of Ljubljana, 1000 Ljubljana, Slovenia; tomaz.ambrozic@fgg.uni-lj.si (T.A.); dusan.kogoj@fgg.uni-lj.si (D.K.); ales.marjetic@fgg.uni-lj.si (A.M.); gasper.stebe@fgg.uni-lj.si (G.Š.); simona.savsek@fgg.uni-lj.si (S.S.); 2 BKR Ingenieurbüro GmbH, 69469 Weinheim, Germany; mozina.jan@gmail.com

**Keywords:** crane rail, terrestrial laser scanning, control measurements

## Abstract

This article presents a method for measuring the geometry of crane rails with terrestrial laser scanning (TLS). Two sets of crane rails were divided into segments, their planes were adjusted, and the characteristic rail lines were defined. We used their profiles to define the positional and altitude deviations of the rails, the span and height difference between the two rails, and we also verified that they complied with the Eurocode 3 standard. We tested the method on crane rails at the hydroelectric power plant in Krško and the thermal power plant in Brestanica. We used two scanning techniques: “pure” TLS (Riegel VZ-400) and “hybrid” TLS (Leica MS50) scanning. This article’s original contribution lies in the detailed presentation of the computations used to define the characteristic lines of the rails without using the numeric procedures from existing software packages. We also analysed the influence of segment length and point density on the rail geometry results, and compared the two laser scanning techniques. We also compared the results obtained by terrestrial laser scanning with the results obtained from the classic polar method, which served as a reference point for its precision.

## 1. Introduction

A crane’s bridge has to move along the crane’s rails with minimum inclination and resistance, as we otherwise encounter the unacceptable wear of wheels and rails. Wrong movements result in expensive repairs, loss of work, and inefficient crane use. The correct position and geometry of the crane needs to be ensured. The condition of the crane rails is monitored by control measurements.

The goal of our research was to discover whether terrestrial laser scanning (TLS, [1,2]) could be used to obtain a point cloud that would provide data for the control measurements of a crane rail’s geometry that would allow us to state with certainty that the construction fulfils the demands set by the Eurocode 3 standard.

TLS measurements were performed on two sets of crane rails, each approximately 50 m long: one in the production hall of the hydroelectric power plant (HPP) in Krško (Figure 3), the other in the thermal power plant (TPP) in Brestanica (Figure 6).

Most of the research carried out so far has focused on railway rails. Most authors have focused on efficient methods for automatically recognising rails from a cloud of points, and creating a three-dimensional (3D) model and trajectories. Babenko [3] presented an algorithm based on the computer vision method. He measured the span between the rails by performing two TLS scans, which he took from a special wagon that moved along the rails. Benito [4] developed a method that automatically created a 3D model of the rails from a point cloud. The author divided the point cloud into multiple equal parts, from which the algorithm automatically recognised the straights on the rails. He created a 3D model of the rails with the least squares and Bézier curve methods. Elberink et al. [5] described the method they used to recognise and model rails from a point cloud captured with a mobile TLS. They solved the problem with the random sample consensus (RANSAC) algorithm. Soni et al. [6] showed the possibility of using a static TLS for determining the geometry of railway rails. They matched various parts of the rails with the standardised rail model. Due to slightly worse precision, the goal of their next study [7] is to improve the precision by trying to optimise the capturing and purification of data and the fitting of the planes. Kopačik and Wunderlich [8] tested the usability of TLS on hydro technical objects [9,10], where they also performed control measurements of crane rails [11]. In their research, they failed to present the precise work methodology, but they did write that TLS was appropriate for such tasks [12]. In their study, Kremen and co-workers [13] tested the possibility of using TLS for the control measurements of crane rails. They scanned the rails from several points, filtered the point cloud, and divided them into segments of the same length. In order to determine the parameters of the crane rails, the rails were approximated by planes. They ascertained that the results obtained through TLS are comparable to the classical measurement method. They proved that TLS could be used for the control measurement of a crane rail’s geometry. They performed over 25 scans from two different points to scan a 73 m long single rail, which was one quarter of the length of the crane rail. This specific study was performed by Kostov [14], Cabaleiro et al. [15], and Asís López et al. [16], who used TLS technology to perform control measurements, and analyse the geometry of steel constructions and calculate their geometric optimisation.

In our research, we attempted to evaluate the influence that segment length and point density have on determining the parameters of a crane rail’s geometry. Based on our analysis, we can determine the length of the segment and the necessary density of the scanned points. When we attempt to ascertain a crane rail’s geometry using the TLS method, these two pieces of data are necessary.

## 2. Measurement Method and Computations

**Figure 1:**
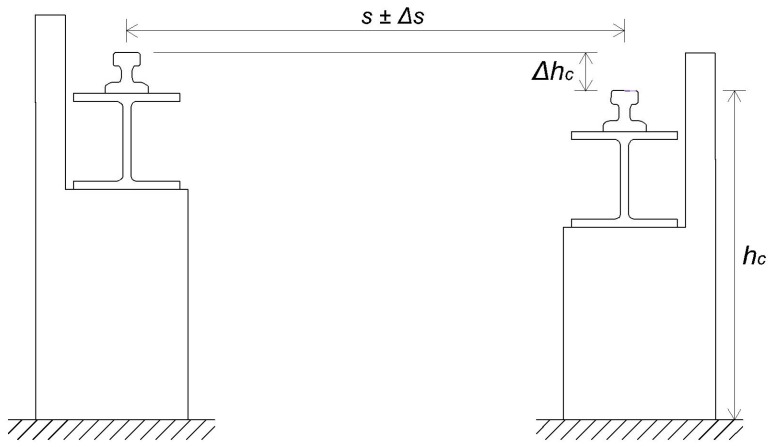
Span tolerance and height difference between the rails [17].

The Eurocode 3 standard addresses the construction of steel constructions. Crane rails need to fulfil the following demands [17]:

- the actual span can deviate from the projected span by a maximum of Δs≤10 mm (Figure 1),
- the height difference $\Delta h_{C}$ between the rails within an individual cross section has to be in line with $\Delta h_{C}\leq s/600$.

The horizontal position and the height of the rails can be measured with various methods:

- alignment and geometric levelling,
- polar method with total station (TPS), and
- terrestrial laser scanning (TLS).

Alignment is a method which allows us to determine the horizontal position of the crane rails [18]. The measurements are performed with a theodolite, a target, and a special ruler with millimetre marks. The height of the rails is defined with the geometric level method, with which the height differences of the rails are defined by levelling their endpoints. In order to perform the measurements, we need to use the lever and the levelling staff.

**Figure 2:**
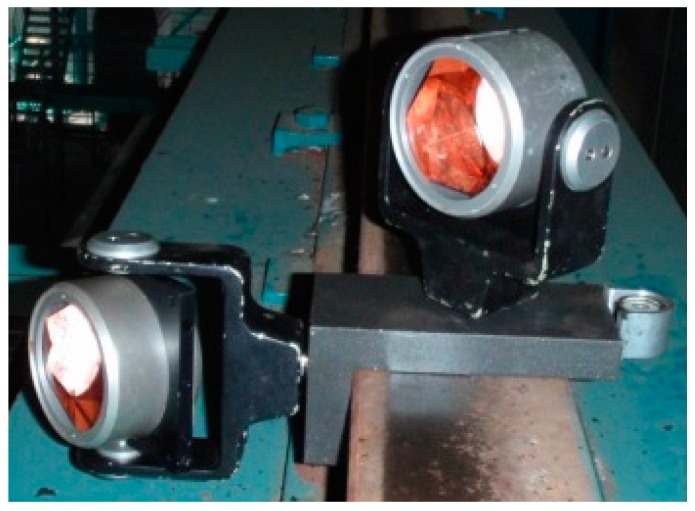
A special platform with two precise prisms.

A TPS measurement can be performed with a precise total station (Leica Geosystems TS30 R1000, Leica Geosystems, Heerbrugg, Switzerland), which is used to measure the horizontal and vertical position of the selected characteristic points. The points on the rail should be measured from a single point, as this ensures homogeneous precision and helps us avoid any mistakes that would occur while establishing a geodetic network. A special platform (Figure 2) with two precise prisms is to be used to signalise characteristic points [19].

TLS [20] helps us to quickly and remotely capture 3D coordinates of groups of points on the rails. The field measurements result in a point cloud. The position of an individual point in the point cloud is defined by polar or rectangular coordinates in the scanner’s coordinate system.

### 2.1. Measuring the Crane Rails in the Machine Room in the HPP in Krško

**Figure 3:**
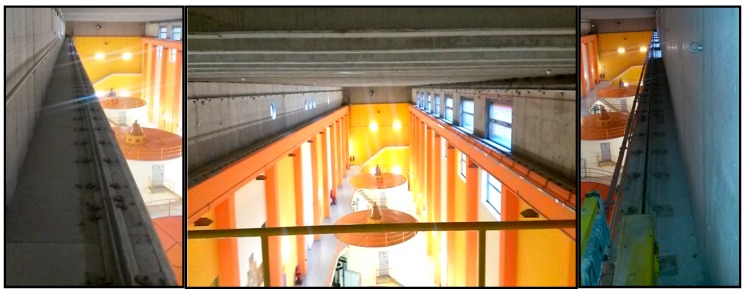
Crane rails in the machine room in the hydroelectric power plant (HPP) in Krško.

Two bridge cranes are located within the machine room’s hall in the HPP in Krško (Figure 3). They are used for installing, overhauling, and performing repairs to electric machinery. The crane rails measure 73 m in length, and the projected span between the rails is 13.1000 m.

**Figure 4:**
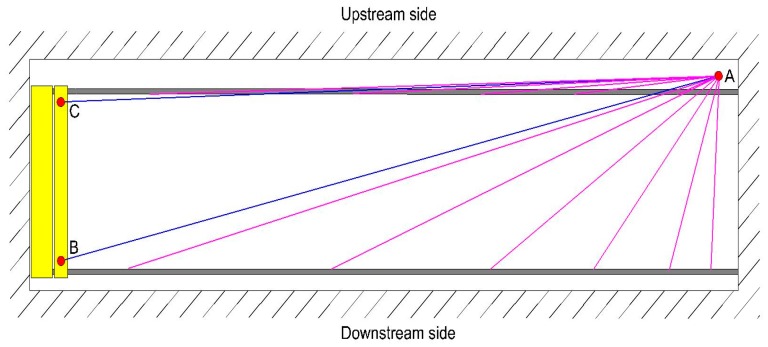
The classic TPS method for measuring crane rails.

The crane rails were measured using both the TPS and TLS methods. The local coordinate system was stabilized with points A, B, and C, which form a triangle (Figure 4). The points served as starting points for the TPS measurement of the rails’ geometry as well as for the TLS measurements.

**Figure 5:**
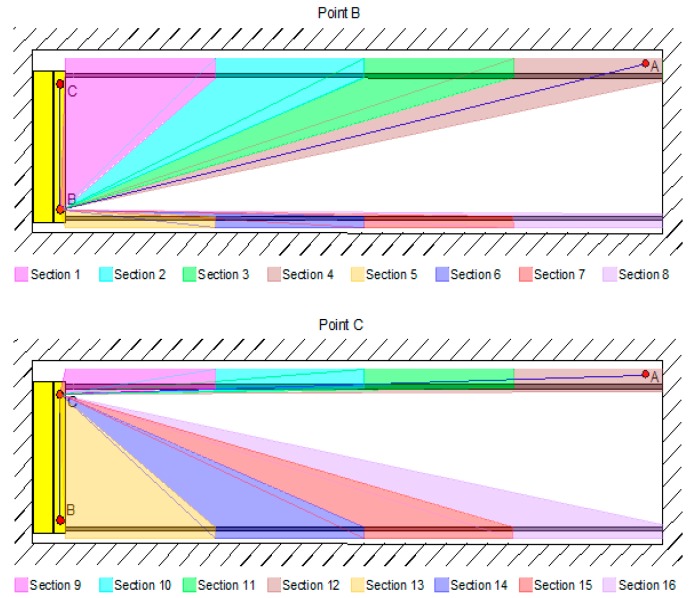
The scans of the sections, from both points.

The geometry of the geodetic network ensured that the rail was seen in its entirety from every point. Points B and C were selected on the crane bridge, while point A was stabilised on the opposite side of the crane rails (Figure 5). The points were signalized with precise GPH1P prisms attached to tripods. We only needed to define the relative position of the rails within the instrument’s coordinate system, which was then transformed into the local coordinate system of the crane rails. Points A, B, and C form the geodetic network defined by tachymetric measurements. For our measurements, we used the Leica Geosystems TS30 R1000 total station. The horizontal directions were measured with seven sets of angles. Also, the slope distances and zenith distances were measured in with seven repetitions and in both faces. For this, we used the Automatic Target Recognition system. The temperature, humidity, and air pressure were measured alongside the instrument for every measuring point. The horizontal positions and heights of the three reference points were calculated individually with the least squares adjustment. The results are shown in Table 1.

#### 2.1.1. TPS Measurement of the Crane Rails

The TPS measurement of the crane rails was performed with the Leica Geosystems TS30 R1000 total station and two precise Leica Geosystems GPH1P prisms attached to platform L [19]. The measurements were carried out from point A (Figure 4). This helped us ensure homogenous and precise measurements. Points B and C served as orientation points. Platform L was set 35 times on each rail, which meant that we created 35 profiles, each measuring 1.8 m.

Every time platform L was set onto the rail, we measured the slope distance, the horizontal direction, and the zenith distance from both faces, twice for each of the prisms. After this, we calculated the average of the two measurements. We calculated the standard deviations of the coordinates for all measured points, for this was how we evaluated the precision of our measurements. From these standard deviations, we calculated the average precision of the span and height differences for all points on the rails, as shown in Table 2.

#### 2.1.2. TLS Measurement of the Crane Rails

The terrestrial laser scanning of the rails was performed with the Leica Geosystems Nova MS50 tachymeter. We scanned from points B and C. In order to ensure a homogenous density of points along the entire rail (Figure 5), each rail was divided into sections, and we scanned the sections with various angular grids (Table 3). We set the angular grids in line with the distance of the instrument from the beginning of each new section.

The Leica Nova MS 50 total station was centred, levelled, and oriented, which enabled us to directly georeference the point clouds into the local coordinate system provided by the previously measured network of points A, B, and C. Direct geo-referencing precision does not depend merely on the precision of placing the instruments and targets, but also on the precision of the coordinates of the given points. When using the direct georeferencing mode, there is no demand to overlap the point clouds. This mode does not depend on the configuration of given points, thus it is best suited for measuring longitudinal objects such as crane rails.

We divided the scans of the rails into 61 equal segments, i.e., we described the crane rails on the basis of 61 profiles; the distance between the profiles was 1 m.

### 2.2. Crane Rail Measurements in the Gas Block Hall in the TPP in Brestanica

**Figure 6:**
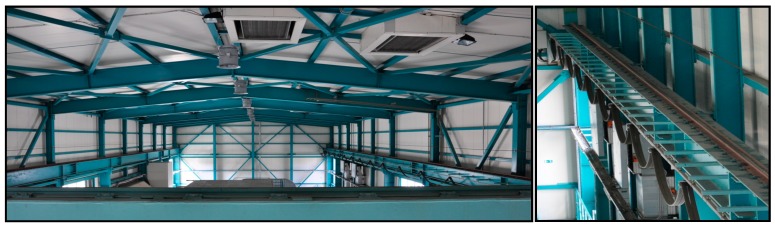
Crane rails in the gas block hall in the thermal power plant (TPP) in Brestanica.

**Figure 7:**
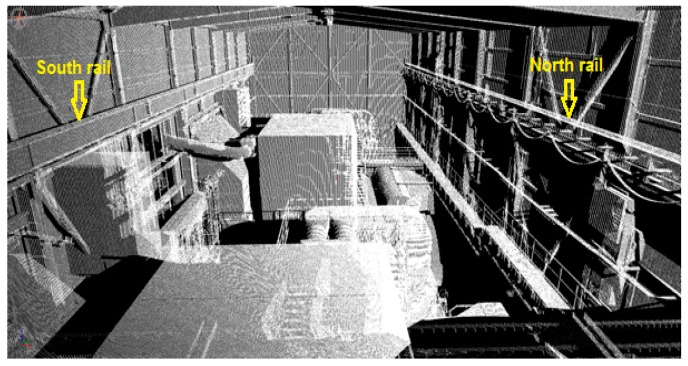
Point cloud in the gas block hall.

The crane rails are located in a hall with two gas turbines (Figure 6 and Figure 7). The rails—which are attached to a steel construction—measure 55.4 m in length, while the projected span between the rails was 19.3000 m.

**Figure 8:**
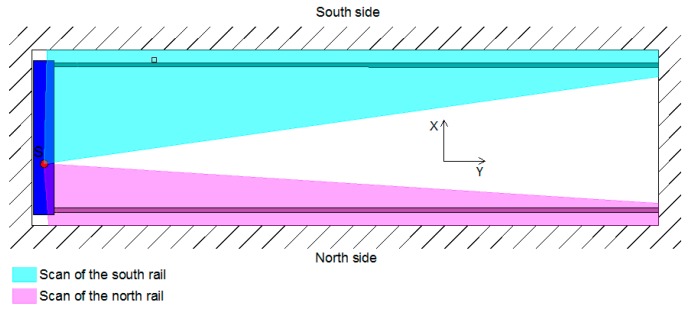
The scan of the rails.

The crane rails were scanned with the RIEGL VZ-400 terrestrial laser scanner. The scanning could only be performed from a single point in the middle of the crane’s bridge (Figure 8). This position ensured that we obtained appropriate results for the inner sides of both rails. The selected scanner height made it possible for us to scan the upper side of both rails. We did not divide the rails into segments as we did in the HPP in Krško; instead, we captured the entire rail with a single measurement. We selected the scanning grid, which equalled a point density of 0.02 m (almost 1’) at the remotest part of the rail. The density of the points on the part of the rails closest to us was very high; however, with distance the density decreased, but remained sufficient for computing the rail geometry (Figure 7).

### 2.3. Treating the Point Clouds and Computing the Rail Parameters

**Figure 9:**
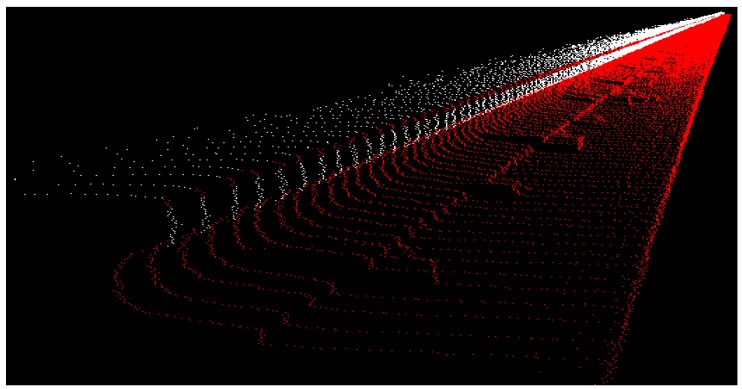
The points that had to be manually eliminated from the scanned point cloud are marked in red.

From the point cloud we manually filtered the points that greatly deviated from the upper or inner side of the rail’s head. The upper surface is 10 cm and 7.5 cm wide, and the side surface 3 cm and 2.5 cm high for the TPP in Brestanica and the HPP in Krško, respectively. As the edge was not sharp, but rounded, we eliminated all of the points that lay on the edge between the upper and inner side during the rough filtering process (Figure 9). The points on the edge that were not removed could cause noise when adjusting the plane. In the future, we are going to further investigate the automatisation of the rough filtering and the partition into an upper and a side plane.

**Figure 10:**
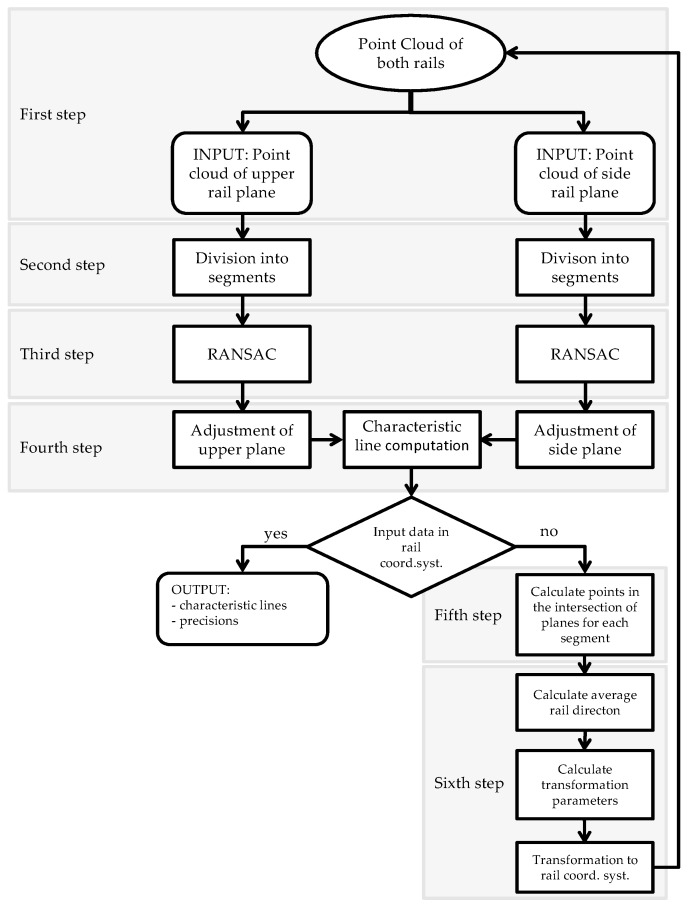
The algorithm used for calculating the point clouds and characteristic lines.

The rail parameters that were compared to the standard were computed in two parts. At first, we computed the characteristic lines of the rails with an algorithm that is schematically shown in Figure 10. Following this, we used the characteristic lines to calculate the span and height differences of the rails.

#### 2.3.1. Treating the Point Clouds and Calculating the Characteristic Rail Lines

**In the first step** (Figure 10) we chose one crane rail within the instrument’s coordinate system as input data. We addressed the point cloud that belonged to the upper panel of the rail’s head separately from the point cloud that belonged to the inner panel of the rail’s head.

**In the second step** (Figure 10) we divided the point cloud into equally long segments.

**In the third step** (Figure 10) we used the RANSAC algorithm [21,22]. We used this algorithm to fine filter and eliminate the roughly misplaced points that could not be defined as points in the vicinity of the rails during the manual filtering process. Each panel of each individual segment was filtered independently. We selected the appropriate tolerance, on the basis of which the algorithm defined the inliers and outliers. The points that were lying within the selected tolerance were defined as inliers and were used as input data in the levelling process.

**In the fourth step** (Figure 10) we adjusted the plane through the scanned points, using the least square method with singular value decomposition (SVD) [23].

The equation of the plane is written as follows:

(1) ax+by+cz+d=0.

We defined the coefficients a, b, c, and d so that the sum of the squares of the orthogonal distances between the points and the adjusted plane was minimal. We minimised the following function:

(2) $$f\left(a,b,c,d\right)=\sum_{i=1}^{n}\frac{{\left(ax_{i}+by_{i}+cz_{i}+d\right)}^{2}}{\left(a^{2}+b^{2}+c^{2}\right)},$$

where $d=-\left(ax_{0}+by_{0}+cz_{0}\right)$.

We assumed that $a^{2}+b^{2}+c^{2}=1$, retransformed function f(a,b,c,d), and found the minimum for the function:

(3) $$f\left(a,b,c\right)=\sum_{i=1}^{n}{\left(a\left(x_{i}-x_{0}\right)+b\left(y_{i}-y_{0}\right)+c\left(z_{i}-z_{0}\right)\right)}^{2},$$

where (a,b,c) represents the normalised vector, $\left(x_{i},\;y_{i},\;z_{i}\right)$ represents the coordinates of the points that represent the point cloud, and $\left(x_{0},\;y_{0},\;z_{0}\right)$ represents the coordinates of the point cloud center.

Matrix A can be written as follows:

(4) $$A=\;\left[\begin{array}{ccc} x_{1}-x_{0} & y_{1}-y_{0} & z_{1}-z_{0}\\ x_{2}-x_{0} & y_{2}-y_{0} & z_{2}-z_{0}\\ \begin{array}{c} \vdots \\ x_{n}-x_{0} \end{array} & \begin{array}{c} \vdots \\ y_{n}-y_{0} \end{array} & \begin{array}{c} \vdots \\ z_{n}-z_{0} \end{array} \end{array}\right],$$

and we split SVD decomposition into $A=U\Sigma V^{T}$. The normalised vector of the plane is located in matrix V. The vector that belongs to the lowest singular value represents the normal of the plane, while the vectors that belong to the greatest singular values span the adjusted plane. The precision of the adjusted plane is calculated from the point cloud, which is orthogonally projected onto the adjusted plane. The misclosure $r_{i}$ is the distance of point *i* from the *adjusted plane*:

(5) $$r_{i}=ax_{i}+by_{i}+cz_{i}+d.$$

The precision of the adjusted plane is described by the estimator $\sigma_{r}$:

(6) $$\sigma_{r}=\sqrt{\frac{\sum_{i=1}^{n}{\left(r_{i}-\bar{r}\right)}^{2}}{n-1}}.$$

This results in two vectors that stretch the plane and the normalised vector. We level each panel in its individual segment. The cross-point of the adjusted planes within an individual segment represents the characteristic line.

The characteristic line of the rail which represents the inner upper edge of the rail head is defined as a cross-section of two planes with the equation:

(7) $$a_{1}x+b_{1}y+c_{1}z+d_{1}=a_{2}x+b_{2}y+c_{2}z+d_{2}.$$

The cross section of these two planes is represented by a line, which runs through point $A\left(x_{0},\;y_{0},\;z_{0}\right)$, and has a directional vector $\vec{s}=\left(f,\;g,\;h\right)$. The equation for this line can be written in the parametric form:

(8) $$x=x_{0}+ft,\;y=y_{0}+gt,\;z=z_{0}+ht.$$

The directional vector of the line is calculated with the vector product of the two normalised planes:

(9) $$\vec{n_{1}}\times \vec{n_{2}}=\left|\begin{array}{ccc} f & g & h\\ a_{1} & b_{1} & c_{1}\\ a_{2} & b_{2} & c_{2} \end{array}\right|.$$

The characteristic lines represent the course of the upper inner edge of the rail’s head, i.e., the line that represents the starting point for calculating the spans and height differences between the rails. The precision of the characteristic line is described with the estimator of the characteristic line $\sigma_{CL}$, and depends on the precision of the adjusted plane of the upper $\sigma_{up}$ and side panels’ surface $\sigma_{sp}$:

(10) $$\sigma_{CL}=\sqrt{\sigma_{up}^{2}+\sigma_{sp}^{2}}.$$

**In the fifth step** (Figure 10) we defined the points on the characteristic line for the individual segments on both rails. We selected one point on each characteristic line of the two rails. The point through which the line runs can be defined in a number of ways. In our example, we chose x=0 or y=0 or z=0 and solved the equation system $a_{1}x+b_{1}y+c_{1}z+d_{1}=0$ and $a_{2}x+b_{2}y+c_{2}z+d_{2}=0$.

**In the sixth step** (Figure 10) we transformed the rails’ point cloud from the coordinate system of the instrument to the coordinate system of the crane rails. The coordinate system of the crane rails is defined by the z axis (vertical), the y axis (runs parallel to the rails and is perpendicular to axis z), and the x axis, which completes the right-handed rectangular coordinate system; the starting point of the coordinate system is arbitrary; the unit is meters.

In order to transform the scanned point cloud from the local scanner to the coordinate system of the crane rails, we needed to ensure three rotations. The translations were arbitrary, while the scale was defined by the measured lengths. The rotations around axes x and y represent the non-horizontal position of the scanner. Some scanners can be levelled (e.g., Leica), and do not need to be rotated around axes x and y. Some scanners have built in inclination sensors (e.g., Riegl), and thus measure the rotations around axes x and y. For rotations around the z axis, we searched for the angle between the y axis of the scanner’s coordinate system and the average direction of the rails.

We used the least squares method to adjust the line through all of the points that we had defined on the characteristic lines of the two rails. The adjusted line represents the average direction of the crane’s two rails. On the basis of the adjusted line, we could calculate the angle of the rotation around axis z (orientational direction). We could also compound Euler’s rotation matrix, which rotates the scanner’s coordinate system into the coordinate system of the crane rail.

In the second iteration we repeated the first, second, and fourth step (the third step was not repeated, as the point cloud has already been finely filtrated).The rails were once again divided into segments of a desired length, the planes were adjusted once again, and the new characteristic lines were calculated.

**Figure 11:**
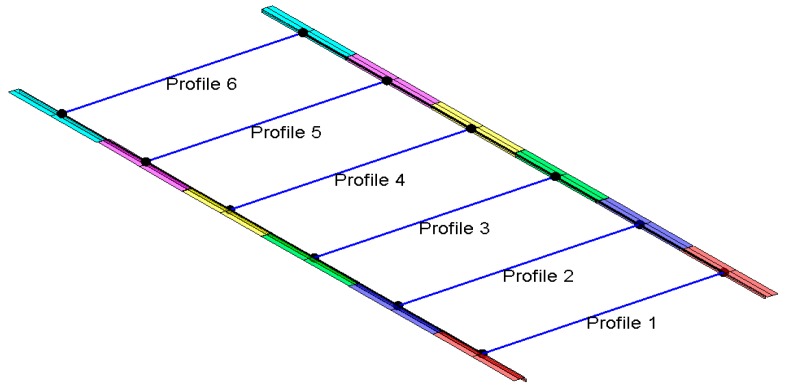
Three-dimensional (3D) presentation of the part of the crane rails with profiles.

The algorithm provided the parameters of the characteristic lines and their precisions for each individual segment. These results also served as input data for the second algorithm, which calculated the deviations, spans, and height differences of the rails (Figure 11).

As we wished to compare the results obtained by scanning with the results obtained through TPS measurement, we transformed the characteristic points of the TPS measurement in the same way as we did the scanned data. This helped us present the data on the position of the rails from both methods in the same coordinate system.

#### 2.3.2. The Computation of the Geometry of the Rails

The geometric parameters of the crane rails were calculated using a second algorithm. The input data were represented by the characteristic lines of the rails, the precision of the adjusted planes, the projected range of the crane rails, and the width of the upper plane of the rail’s head. The information on the projected span and the width of the upper plane of the rail can be found in the project documentation for the treated crane rails. According to the Eurocode 3 standard, the projected span is defined between the axes of the two rails. In our research, we calculated the span between the inner side planes of the head of the two rails. Thus, we needed to take into account the width of the upper side of the head of the rail if we wished to ascertain the difference between the projected and the actual range.

We computed the characteristic point for each pair of segments on the left and right rail. The profile represented the x-z plane in the centre of each segment. Each pair of segments has one profile. We calculated the characteristic segment points as a cross-section of the profile, and the characteristic lines by entering the stationary of the individual y profile into the equation for the line. We used the characteristic points to define the course of the crane rails, i.e., the ranges, the height differences, deviations from projected line, and the straightness and parallelism of the rails.

**The span** on an individual profile equals the difference of the coordinates x of the characteristic points of this profile. The precision of the span, described by the standard deviation of span $\sigma_{s}$, depends on the precision of the definition of the side plane of the first $\sigma_{sp_{1}}$ and second $\sigma_{sp_{2}}$ rail:

(11) $$\sigma_{s}=\sqrt{\sigma_{sp_{1}}^{2}+\sigma_{sp_{2}}^{2}}.$$

**The positional deviations** of the characteristic points from the projected positions are defined by the referential lines of the crane rails. The referential lines are lines in the x-y plane, which are oriented in the direction of the y axis and distanced by the projected span at which the width of the rails needs to be taken into account. The centre between the referential lines coincides with the average of the x axis coordinates for all characteristic points. The positional deviation is the distance between the characteristic point and the referential line in the x-y plane.

**Height differences** between the profiles on the left and right rail are calculated as the difference of the coordinates z of the characteristic points. The precision of the height difference between the rails, described by the standard deviation of the height difference $\sigma_{\Delta h_{C}}$, depends on the precision of the definition of the upper plane of the left $\sigma_{up_{1}}$ and right $\sigma_{up_{2}}$ rail:

(12) $$\sigma_{\Delta h_{C}}=\sqrt{\sigma_{up_{1}}^{2}+\sigma_{up_{2}}^{2}}.$$

**The vertical deviations** of the characteristic points are computed in relation to the referential level plane, which is defined as the average of the height coordinates of all characteristic points.

## 3. Results and Analysis

### 3.1. Results: Spans and Height Differences

#### 3.1.1. TPS Measurement of the Crane Rails in the HPP in Krško

Particularly for the HPP in Krško, the standard requires that the actual span of the rail has to be within 13.0900 m and 13.1100 m. Taking into account the designed span of the crane rails, the permitted height difference in an individual cross-section or profile is 21.8 mm.

**Figure 12:**
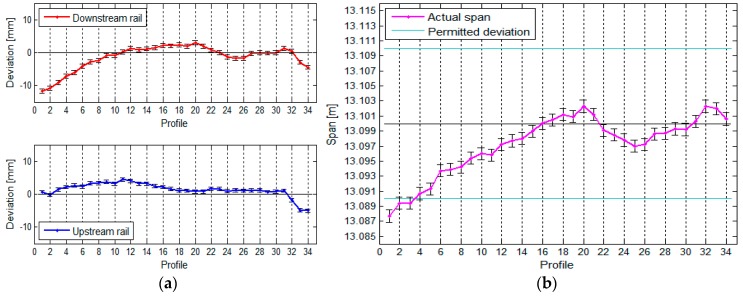
(**a**) The rails and their positional deviations, and (**b**) the span between the rails, with the standard deviations, for the crane in the HPP in Krško measured with TPS.

The TPS measurement of the crane rails provided us with the following results, which are presented in graphic form. The rails, their horizontal deviations, and the span between the rails are shown in Figure 12.

Figure 12 shows that the crane rails are neither parallel nor straight. From the way we calculated the span, we can conclude that the precision of defining the span is constant along the entire crane rail (see Table 2).

**Figure 13:**
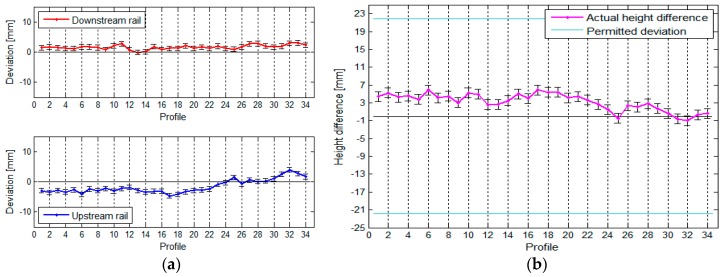
(**a**) The vertical deviations and (**b**) the height differences between the rails, with their standard deviations, for the crane in the HPP in Krško measured with TPS.

The vertical deviations of the crane rail and the height differences between the rails are shown in Figure 13.

Figure 13 reveals that the downstream rail is slightly higher than the upstream rail.

#### 3.1.2. TLS measurement of the crane rails in the HPP in Krško

**Figure 14:**
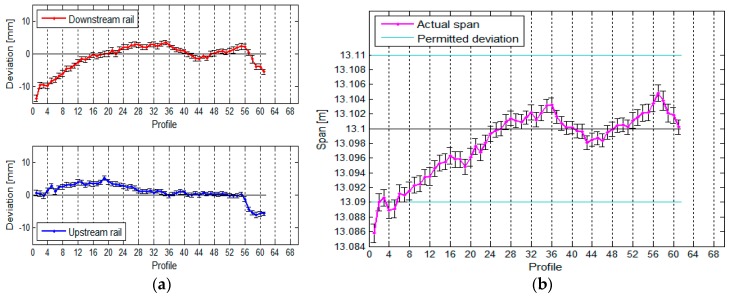
(**a**) The positional deviation of the rails and (**b**) the span between them, with their standard deviations, for the crane in the HPP in Krško measured with TLS.

**Figure 15:**
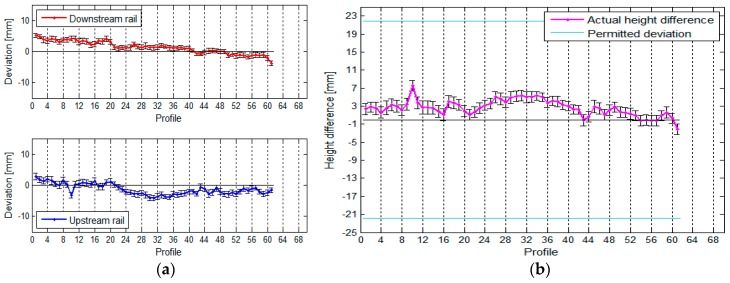
(**a**) The vertical deviations and (**b**) the height differences between the rails, with their standard deviations, for the crane in the HPP in Krško measured with TLS.

The TLS measurement of the crane rails provided us with results that are presented here in graphic form. Figure 14 shows the positional deviation and span between the rails, while Figure 15 shows the vertical deviations and height difference between the rails.

For each profile, we defined the standard deviation of the side plane for the first and second rail $\sigma_{sp_{1}}$ and $\sigma_{sp_{2}}$ as well as the standard deviation of the span between the rails $\sigma_{s}$. The average standard deviation of the positional deviations is less than 1 mm for both rails, while the average precision of the spans measured in at 1.0 mm.

Alongside the vertical deviations and height differences we also calculated the standard deviation of the upper plane of the first and second rails $\sigma_{up_{1}}$ and $\sigma_{up_{2}}$ as well as the standard deviation of the height differences $\sigma_{\Delta h_{C}}$ for each profile. The average standard deviation of the upper plane is less than 1 mm for both rails, while the average standard deviation of the height differences measured in at 1.2 mm.

#### 3.1.3. TLS Measurement of the Crane Rails in the TPP in Brestanica

**Figure 16:**
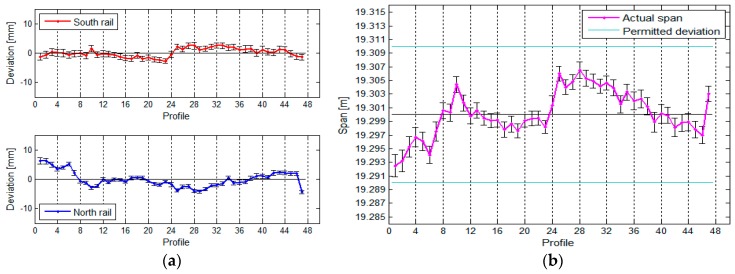
(**a**) The rails’ deviation and their positions, and (**b**) the span between the rails, with the standard deviations, for the crane in the TPP in Brestanica measured with TLS.

If we want to state that the rails are in line with the standard, the actual span of the crane rails in the TPP in Brestanica has to be between 19.2900 m and 19.3100 m. Based on the designed span, we can also define the highest possible permitted height difference between the rails within an individual profile, and this amounts to 32.2 mm. We have divided each rail into 47 segments, each measuring 1 m in length, which means that each rail has 47 profiles. The position of the rails and the span between them can be seen in Figure 16.

The average standard deviation of the positional deviations of the south rail is 1.0 mm, and that of the north rail is 0.6 mm (due to the slightly worse geometry of the rail in relation to the instrument). The standard deviation of the calculated spans is 1.2 mm.

**Figure 17:**
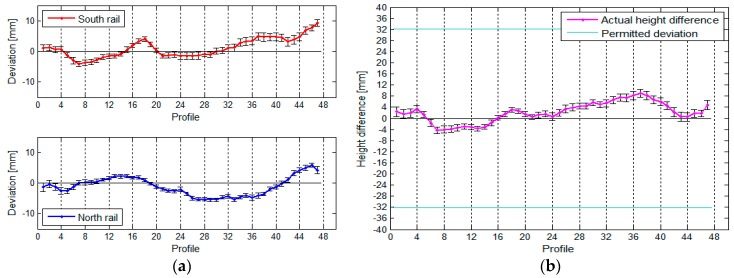
(**a**) The vertical deviations and (**b**) the height differences between the rails, with their standard deviations, for the crane in the TPP in Brestanica measured with TLS.

The vertical deviations and height differences between the rails are shown in Figure 17.

The average standard deviation of the upper south rail is 1.0 mm, and that of the north rail is 0.7 mm. The average standard deviation of the height differences is 1.2 mm.

### 3.2. The Influence of Segment Length on the TLS Measurement Results

**Figure 18:**
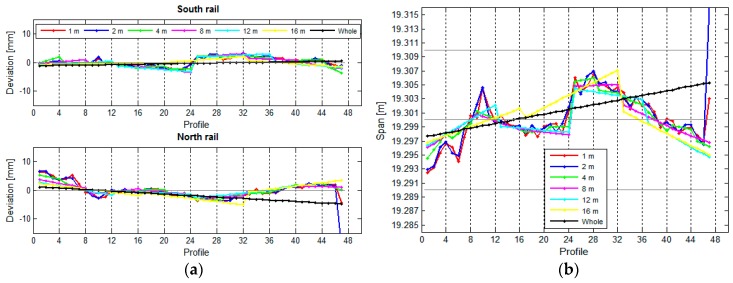
(**a**) The positional deviations and (**b**) the spans in relation to the segment length.

**Figure 19:**
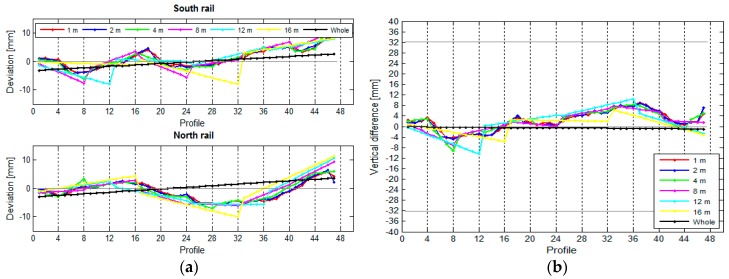
(**a**) The vertical variances and (**b**) the height differences in relation to the length of the segment.

In our research, we have used the results obtained for the crane rails in the TPP in Brestanica. We were interested in how the length of the rail segment influenced the deviation, span, and height differences between the two rails. We divided the two rails into segments measuring 1 m, 2 m, 4 m, 8 m, 12 m, and 16 m, and we have also treated the rail as a whole, as a single unified segment. We have calculated the deviations, spans (Figure 18), and height differences (Figure 19) for these segments.

Figure 18 reveals that the position of the rails is defined correctly regardless of the length of the segment. A shorter segment results in higher resolution. The position of the rails will be defined just as well with longer segments, but with lower resolution. The same holds true for elevation (Figure 19).

**Figure 20:**
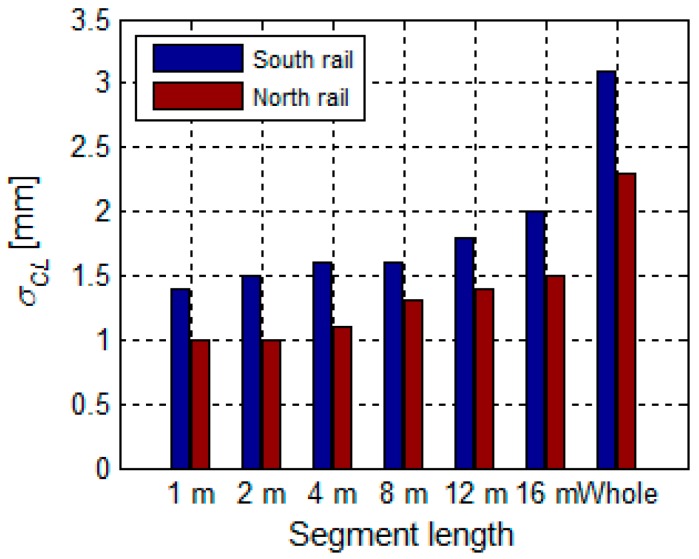
Precision of the characteristic lines in relation to the length of the segment.

The longer the segment, the lesser the chances for a detailed measurement of the deviations, spans, and height differences of the rail. Figure 20 shows that the standard deviation of the characteristic line $\sigma_{CL}$ increases with the length of the segment.

### 3.3. The Influence of the Density of Scanned Points on TLS Measurements

We performed tests for rail measurements at the TPP in Brestanica. We have selected the basic model, at which the segment length measured 1 m. The scanning resolution was set to 0.02 m at the remotest part of the rail, which correspond to an angle of 1’. The obtained density for the south rail (Figure 8) was $1.4\times 10^{4}$ points per meter at the beginning of the rail and $4\times 10^{2}$ points per meter at the remotest part of the rail. As for the north rail (Figure 8), the densities at the beginning and at the end of the rail were $4\times 10^{5}$ and $3.5\times 10^{2}$ points per meter, respectively.

**Figure 21:**
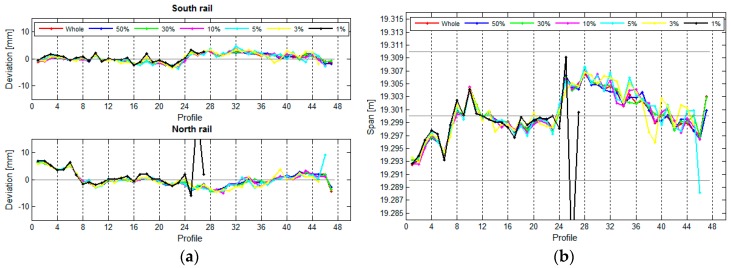
(**a**) The positional deviations of the two rails and (**b**) the spans in relation to the point density.

**Figure 22:**
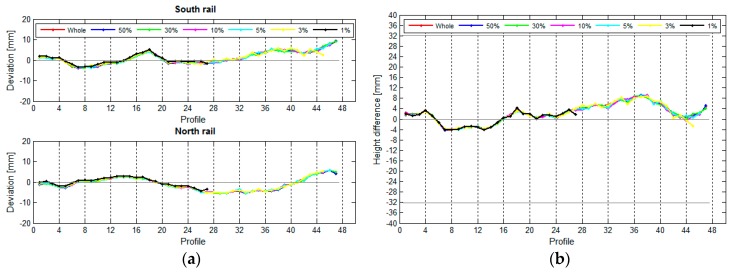
(**a**) The vertical deviations and (**b**) the height differences in relation to point density.

We have ascertained the influence of random subsampling at 50%, 30%, 10%, 5%, 3%, and 1% of the points from the individual rail plane. Figure 21 and Figure 22 show the positional deviations of the two rails, as well as their spans, vertical deviation, and height differences in relation to the density of the rail points.

Once we use 3% of the points, the rail can be defined up to the 45th profile (out of 47), while if we use 5% of the points, it can be defined up to the 46th profile. We can also notice that if we use 1% of the points, the rail can be defined only until the 27th profile, as in the last part of the rail—in comparison to the first part of the rail—the density of the points is reduced (due to the scanning geometry). Thus, we established that the results become unreliable when actual point density is less than 30 points per meter. If we use only 1% of all of the points, we do not have a sufficient amount of points in the last part of the rail to determine the adjusted plane. Table 4 shows certain statistical parameters of the positional deviations of the crane rails once we remove a certain percentage of the points. The base is represented by the computation of the deviations when all points are used.

We can see that the vertical deviations are almost identical at all point densities, and these result in almost identical height differences. Table 4 shows that the average values of the vertical deviations differ by less than 1 mm (in relation to the deviations calculated from all points) even after only 1% of the points have been used.

### 3.4. Comparison of TLS and TPS Measurements

The geometry of the crane rail in the HPP in Krško was defined with the use of two surveying methods—with the TPS polar measurement method and with the TLS method—see Section 3.1.1 and Section 3.1.2. In order to ensure comparability, the measurements from both methods were transformed to a common local coordinate system of the crane rails (see Section 2.3.1). For the TLS measurement, the rails were divided into 34 segments measuring 1.8 m each, the same number of segments as used in the TPS measurement. The comparison of the results obtained by the two methods is presented numerically and graphically.

**Figure 23:**
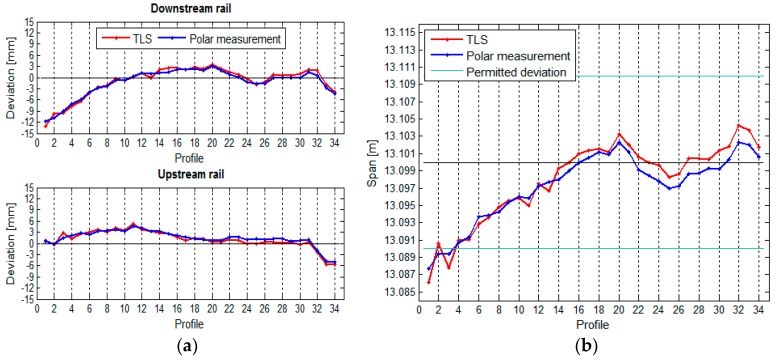
(**a**) Comparison of the horizontal deviations and (**b**) the spans between the rails.

**Figure 24:**
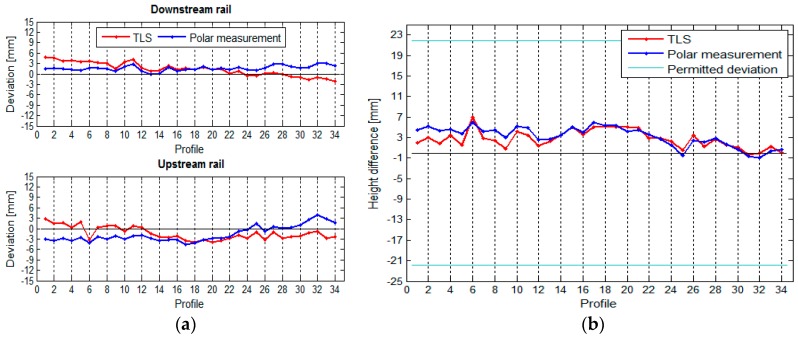
(**a**) Comparison of the vertical deviations and (**b**) height differences between the rails.

We can see that the horizontal (Figure 23) as well as the vertical (Figure 24) position of the crane rails is similar when measured by both TPS and TLS. We have noticed that the choice of method does not show an important influence on the measurement of the span of the vertical differences between the rails. The inclination of the TLS profile with respect to TPS exists. It is the consequence of the TLS’s inclination sensors, which are not accurate enough. The effect, however, has small influence on the height differences, since the span is significantly shorter than the length of the rails.

Table 5 shows the comparison of the maximum, minimum, and median differences of the horizontal and vertical deviations and spans between the rails calculated by both measurement methods.

## 4. Discussion and Conclusions

In our research, we presented the TLS control measurements method for two different crane rails. Similar to Kremen et al. [13], we approached the process of defining the geometry of the crane rails by dividing the point cloud into segments of the same length. We used two scanning techniques: “pure” TLS (Riegel VZ-400) and “hybrid” TLS (Leica MS50) scanning. The original contribution of this article lies in the detailed presentation of the computations for defining the characteristic lines of the rails independently of the numeric procedures found in existing software, the analysis of the influence of the length of the segment, and the density of points on the rail geometry results. The results obtained with the TLS measurements were compared to the referential results obtained with the TPS method, which we have developed ourselves [19].

Research has shown that terrestrial laser scanning is a suitable technology for defining the geometry of straight crane rails with flat surfaces up to 100 m long. TLS measurements provide up to standard precision for defining the parameters of the crane rails. While measuring and calculating, one has to take into account the specific criteria i.e., fulfil the appropriate demands.

During the scanning process, the geometry of the scanning is also important. The rails have to be scanned under sufficiently low incidence angles, and the point density should remain as consistent as possible along the entire length of the rail. Apart from the scanning geometry, the precision is also influenced by the length of the segment. The length of the segment of the rail, defined in the process of treating the point clouds, has a direct influence on defining the parameters of the rail geometry. The rails have to be divided into segments. The segments should not measure more than 2 m in length, but for best results we suggest that they are 1 m long. This helps us describe the position of the crane rails in sufficient detail (Figure 19).

The density of the scanned points has practically no influence on the geometry parameters of the crane rails. We have ascertained that we can use only 5% of the points from the point cloud without significant changes in the results. When even smaller subsamples are used, it is impossible to adjust the planes through the upper and side planes of the rails in the parts with the lowest point density. Taking the scan results into account, we can conclude that the only condition linked to the point density is that each segment needs a sufficient number of points for adjusting the planes. A density of at least 30 points per meter of the rail is essential to obtain accurate results. The relatively poor precision of the individual scanned point (which we compensate for with a relatively high number of measurements) does not significantly influence the results. This realisation is most welcome, for we do not need a great density when scanning the object. This fact is of great importance when scanning the crane rails. Due to the unfavourable geometry of the rail (as we are dealing with a linear object) and the manner of scanning, we have obtained a scarce point cloud on the parts of the rails that are the furthest away from our measuring point.

Our results have proven that the differences in the horizontal and vertical parameters of the crane rail geometry when measured by TLS and TPS are small enough for us to state that the results obtained with the TLS method are comparable to the results obtained by the TPS method. When we compared the precision of the spans and height differences, we ascertained that the differences are minute, as they amount to 0.2 mm in favour of TPS measurements. Taking into account two important criteria—the geometry of scanning and the length of the rail segment—we can successfully use the TLS method to define the geometry of the crane rails, which is in line with the standardised procedures.

## Figures and Tables

**Table 1:** The adjusted coordinates of the network points in the local coordinate system, with their precisions.

Point	*x* [m]	*y* [m]	*H* [m]	$\sigma_{x}$ [mm]	$\sigma_{y}$ [mm]	$\sigma_{H}$ [mm]
A	1010.4798	989.7332	100.5468	0.1	0.1	0.5
B	949.7709	1012.4671	101.2770	0.1	0.1	0.3
C	955.9898	1022.4994	101.3517	0.1	0.1	0.3

**Table 2:** Average precision of the coordinates, i.e., the precision of the deviations, spans, and height differences of the rails.

Rail	$\sigma_{\Delta x}$ [mm]	$\sigma_{\Delta H}$ [mm]	$\sigma_{s_{d}}$ [mm]	$\sigma_{\Delta h_{c}}$ [mm]
Downstream rail	0.6	0.8	0.8	1.1
Upstream rail	0.5	0.8		

**Table 3:** The horizontal and vertical angular scanning grid, the number of captured points on an individual section, and the time necessary to capture the points on an individual section.

Section	Horizontal Angular Grid [gon]	Vertical Angular Grid [gon]	Number of Captured Points	Scanning Time [min]
Point B				
1	0.0445	0.0089	120,537	9
2	0.0207	0.0041	78,849	6
3	0.0126	0.0025	41,731	3
4	0.0113	0.0023	35,585	3
5	0.0053	0.0400	185,645	12
6	0.0053	0.0053	85,311	3
7	0.0445	0.0445	46,285	3
8	0.0106	0.0106	63,406	3
Point C				
9	0.0646	0.0129	83,791	3
10	0.0192	0.0038	13,382	1
11	0.0127	0.0025	12,105	1
12	0.0285	0.0057	5592	1
13	0.0430	0.0086	66,857	6
14	0.0273	0.0055	72,238	6
15	0.0187	0.0037	55,157	3
16	0.0110	0.0022	48,617	3

**Table 4:** Numeric representation of the deviation differences when removing points.

% of Subsampled Points	50%		30%		10%		5%		3%		1%	
Rail	S	N	S	N	S	N	S	N	S	N	S	N
Positional deviations												
Max. [mm]	0.9	1.7	1.3	1.4	1.5	1.8	2.5	7.2	2.9	2.4	2.8	26.7
No. of diff. >1.0 mm	0	1	2	1	2	3	12	6	9	10	5	10
No. of profiles	47	47	47	47	46	46	46	46	45	45	27	27
Aver. [mm]	0.2	0.3	0.3	0.3	0.3	0.4	0.7	0.6	0.6	0.7	0.7	2.0
±St. dev. [mm]	±0.2	±0.3	±0.3	±0.3	±0.3	±0.4	±0.6	±1.1	±0.6	±0.7	±0.6	±5.0
Vertical deviations												
Max. [mm]	0.6	0.8	1.1	0.8	1.2	0.7	1.1	0.7	4.1	1.1	1.3	1.8
No. of diff. >1.0 mm	0	0	1	0	1	0	1	0	3	1	4	2
No. of profiles	47	47	47	47	46	46	46	46	45	45	27	27
Aver. [mm]	0.1	0.1	0.2	0.1	0.3	0.2	0.2	0.2	0.6	0.4	0.7	0.7
±St. dev. [mm]	±0.2	±0.1	±0.3	±0.1	±0.3	±0.2	±0.2	±0.2	±0.6	±0.2	±0.3	±0.3

**Table 5:** The comparison of horizontal and vertical parameters of the crane rail geometry, performed by both measurement methods.

	Comparison of the Downstream Rail Deviation		Comparison of the Upstream Rail Deviation		Comparison of the Span and Vertical Differences	
	Δx [mm]	ΔH [mm]	Δx [mm]	ΔH [mm]	Δs [mm]	$\Delta h_{C}$ [mm]
Max.	1.4	4.5	1.3	5.9	2.2	2.4
Min.	0.0	0.0	0.1	0.1	0.2	0.0
Ave.	0.6	1.8	0.6	2.5	1.1	1.1
